# Tregs in transplantation tolerance: role and therapeutic potential

**DOI:** 10.3389/frtra.2023.1217065

**Published:** 2023-08-30

**Authors:** Alexandra Cassano, Anita S. Chong, Maria-Luisa Alegre

**Affiliations:** ^1^ Department of Medicine, University of Chicago, Chicago, IL, United States; ^2^ Department of Surgery, University of Chicago, Chicago, IL, United States

**Keywords:** Tregs (regulatory T cells), Foxp 3, transplantation, tolerance, alloimmunity, acute rejection (AR)

## Abstract

CD4^+^ Foxp3^+^ regulatory T cells (Tregs) are indispensable for preventing autoimmunity, and they play a role in cancer and transplantation settings by restraining immune responses. In this review, we describe evidence for the importance of Tregs in the induction versus maintenance of transplantation tolerance, discussing insights into mechanisms of Treg control of the alloimmune response. Further, we address the therapeutic potential of Tregs as a clinical intervention after transplantation, highlighting engineered CAR-Tregs as well as expansion of donor and host Tregs.

## 1. Introduction

Regulatory T cells (Tregs) are required to prevent autoimmunity and have a suppressive role on effector T cells (1, 2). The transcription factor forkhead box P3 (Foxp3) is expressed by Tregs and is indispensable for the Treg suppressive phenotype (3–6), with mutations in Foxp3 resulting in Immunodysregulation Polyendocrinopathy Enteropathy X-linked (IPEX) syndrome (7–9), although recent evidence indicates that microbiota-peripherally induced Tregs (pTregs) are able to retain some functionality in the absence of Foxp3 expression (10). Tregs control the T cell response to self-antigen, suppressing auto-reactive T cells and protecting from catastrophic autoimmunity (11–14). Regulatory CD8^+^ T cells have been characterized, such as CD8^+^ Qa-1-restricted suppressor cells, which have also been implicated in self-tolerance and allograft acceptance (15, 16), and CD8^+^ Foxp3^+^ regulatory cells, which have been described in both cancer and transplantation contexts (17–22). However, these regulatory subsets are less characterized, and this review article will focus on the role of CD4^+^ Foxp3^+^ Tregs in transplantation tolerance.

### 1.1. Treg subsets

Regulatory T cells are a heterogenous population and are often classified based on their origin. Tregs that develop in the thymus, termed tTregs (thymic Tregs) previously referred to as nTregs (natural Tregs), constitutively express Foxp3 and arise from either CD25^+^ or Foxp3^low^ thymic progenitors during negative or positive selection, respectively (23–25). Tregs are often classified as CD3^+^ CD4^+^ Foxp3^+^ CD25^high^ CD127^low^ cells, since Foxp3 and CD127 expression are inversely correlated (26). Thymically derived tTregs make up the majority of the population of Tregs and are indispensable for controlling autoimmunity since they are reactive for self-antigens (2, 5, 24, 27).

Tregs can also develop from CD4^+^ conventional T cells (Tconvs) in the periphery in response to non-self-antigens. These Tregs are termed pTregs and are generated upon strong TCR activation in a tolerogenic environment, where the presence of IL-2, TGFβ and retinoic acid favors pTreg differentiation (24, 27–29). Induced Tregs generated *in vitro*, termed iTregs, are functionally similar to pTregs and require comparable conditions for generation (24, 27–29).

### 1.2. Phenotypic stability of Tregs

Continued expression of Foxp3 is required for the maintenance of the murine Treg phenotype and suppressive function (30), and both tTregs and pTregs adopt a memory T cell inflammatory phenotype upon cessation of Foxp3 expression (31). Inflammatory environments such as infection or autoimmunity can impair Treg stability, exacerbating disease progression. For instance, corneal inflammation following herpes simplex virus type 1 (HSV-1) infection led to a loss of Foxp3 expression in Tregs, which became pathogenic, producing IFNγ and driving stromal keratitis (32). In an autoimmune context, nTregs lost their Foxp3 expression and suppressive ability following co-culture with synovial fibroblasts from collagen-induced arthritic mice (33). On the other hand, certain inflammatory stimuli can enhance Treg stability, with type I interferon (IFN) augmenting Foxp3 acetylation and improving transplant survival (34).

## 2. Mechanisms of Treg suppression

**Figure 1:**
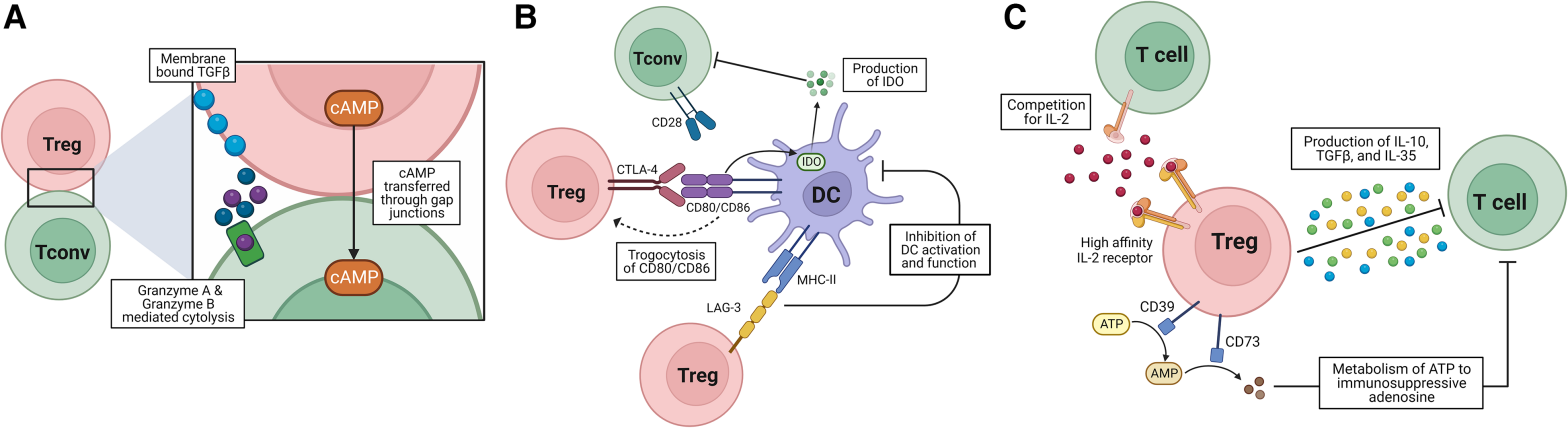
Mechanisms of Treg suppression. (**A**) Cell-contact-dependent interactions with T cells. Tregs can mediate suppression via transferring cAMP through gap junctions, membrane bound TGFβ, or the release of cytotoxic Granzyme A and Granzyme B. (**B**) Cell-contact-dependent interactions with APCs. Tregs express CTLA-4 which outcompetes CD28 on T cells for binding to CD80/CD86 and can mediate trogocytosis of CD80/86 as well as induce DC production of immunosuppressive IDO. LAG-3 binding to MHC-II on APCS inhibits their activation and function, impeding the ability of APCs to activate T cells. (**C**) Cell-contact-independent environmental perturbations. Tregs secrete immunosuppressive cytokines IL-10, IL-35 and TGFβ. Constitutive expression of the high affinity IL-2 receptor allows Tregs to outcompete T cells for IL-2 acting as a cytokine sink. Tregs increase environmental levels of immunosuppressive adenosine via CD39 and CD73 metabolism of ATP and AMP.

Tregs can constrain immune responses through a variety of mechanisms that are contact-dependent and contact-independent. This review will focus on the role of Tregs in suppressing Tconvs, as Tconvs are known drivers of transplant rejection (35–37), although Tregs are also capable of mediating suppression of B cells, NK cells and NKT cells (38–40). Treg suppression mechanisms can be broadly categorized into three types: contact-dependent interactions with antigen-presenting cells (APCs), contact-dependent interactions with T cells, and contact-independent perturbations of the surrounding environment that alter cell metabolism or the cytokine milieu (Figure 1). Tconv proximity to Tregs following transplantation is promoted by Treg-produced chemokines; Foxp3 can bind to the promoter region of CCL3 and CCL4, leading to their increased production after TCR stimulation and the recruitment of CCR5^+^ Tconvs. CCL3-deficient Tregs are significantly less protective of islet allograft rejection (41), suggesting that this contributes to the ability of Treg suppression of graft-reactive Tconvs.

### 2.1. Contact-dependent suppression mechanisms

One of the major methods of contact-dependent Treg-mediated suppression of Tconv proliferation and activation is inducing the upregulation of cell surface inhibitory receptors on Tconvs, which typically occurs indirectly by inhibiting APC activation. For instance, Tregs constitutively express the inhibitory receptor cytotoxic T-lymphocyte associated antigen-4 (CTLA-4) (42). CTLA-4 can outcompete the binding of the activating co-receptor CD28 to their shared ligands, CD80 and CD86, and also downregulate APC expression of CD80 and CD86 via trogocytosis, leaving the APCs less able to activate Tconvs (43–46). Importantly, the interaction between CTLA-4 on Tregs and CD80 and CD86 promotes dendritic cell (DC) expression of indoleamine-2,3-dioxygenase (IDO), a tolerogenic enzyme involved in tryptophan metabolism (47). The expression of IDO on DCs has been shown to inhibit T cell activation and proliferation (48–50), and overexpression of IDO on DCs prolonged murine intestinal allograft survival and expanded Tregs (51). In the same vein, Tregs express lymphocyte activation gene-3 (LAG-3) that binds to MHC-II and contributes to their suppressive activity, possibly by inhibiting DC activation, although this mechanism is less well understood (52–54).

Tregs can also act more directly on target cells themselves, driving apoptosis or suppression of target cells in a contact-dependent manner. Tregs are able to induce cell death through their expression of Granzyme A and Granzyme B (55–59). Tregs are also capable of inducing target cell death through activation of the TNF-related apoptosis inducing ligand (TRAIL)/Death Receptor 5 (DR5) pathway. This mechanism has been implicated in allograft survival as DR5-blocking antibodies prevented skin allograft survival in mice (60). Tregs can also express membrane-bound TGFβ on their cell surface and this may be an avenue of cell-cell contact-mediated Tconv suppression (61). Tregs are also able to enact metabolic changes in target cells to suppress proliferation and activation. For instance, Tconvs exhibit an increase in cyclic adenosine monophosphate (cAMP) and cAMP response element modulator (CREM) expression during cell contact-dependent Treg suppression via transfer of cAMP through gap junctions and this increased cAMP is necessary for inhibition of proliferation and IL-2 expression in target effector T cells (62, 63).

### 2.2. Contact-independent suppression mechanisms

Contact-independent mechanisms of suppression rely on Tregs driving a tolerogenic environment, therefore still requiring close proximity of Tregs to their target cells. For instance, Tregs can suppress Tconvs through secretion of the immunosuppressive cytokines IL-10 (64–68), IL-35 (69–72), and TGFβ (73, 74). Tregs also change the environmental cytokine concentration by acting as an “IL-2 sink” and outcompeting nearby Tconvs for IL-2 through expression of CD25, the high affinity IL-2 receptor α chain (64, 75, 76). Tregs also express the ectonucleotidases CD73 and CD39, which confer suppressive ability to Tregs by mediating the conversion of ATP and ADP to AMP then further to adenosine, thus modulating the extracellular adenosine content (77–80). Increased extracellular adenosine promotes a tolerogenic environment, impairing inflammatory cytokine production by DCs, while ATP has been shown to act as a pro-inflammatory signal (78–81). Tregs are therefore able to mediate bystander regulation in an antigen-agnostic manner; pre-treatment of recipient mice with donor alloantigen and anti-CD4 antibody generated Tregs that were not only sufficient to prevent the rejection of donor-matched skin allografts, but were also capable of preventing rejection of a 3rd party graft if the Tregs were reactivated with the tolerizing alloantigen prior to transplantation (82). Another mechanism through which Tregs are capable of promoting tolerance is through recruitment of other tolerogenic cells. Tregs have been found to recruit mast cells to skin allografts through production of IL-9, and this recruitment was indispensable for long-term tolerance in a costimulation blockade (αCD154) + donor-splenocyte transfer (CoB + DST)-mediated murine allograft tolerance model (83).

Foxp3^+^ Tregs can also secrete small extracellular vesicles (sEVs) that have Nrp1 in their membrane. Nrp1 in Treg-derived sEV promoted skin transplant tolerance and mice treated with Nrp1-KO Treg-derived sEV had decreased graft survival. Nrp1 expression in Tregs is associated with increased suppressive function and is necessary for phenotypic stability. Nrp1^+^ Treg-derived sEVs transferred to graft recipients resulted in significantly improved graft survival compared to Nrp1^−^ sEVs or no sEVs. The presence of Nrp1^+^ sEVs skewed macrophages to a more tolerogenic phenotype in the graft highlighting the role of Tregs in promoting an environment permissive of allograft survival (84).

## 3. The fate of Tregs after transplantation

### 3.1. Treg proliferation and accumulation in the graft after transplantation

It has been well established than an increased ratio of Tregs:Tconvs is observed in tolerance, and that this high ratio is likely necessary for tolerance to occur/be maintained. After transplantation of an F1 Balb/c x B6 murine heart expressing the fusion protein 2W:OVA, endogenous donor-reactive Tregs (identified as 2W:I-A^b^ tetramer-binding Foxp3^+^ CD4^+^ T cells) accumulated in the spleen at comparable rates in both rejection and CoB + DST-mediated tolerance (85). In this model, during CoB + DST-mediated tolerance, endogenous donor-reactive Tconvs (identified as 2W:I-A^b^ tetramer-binding Foxp3^−^ CD4^+^ T cells) proliferated less than they did in untreated hosts during acute rejection (85). This unequal expansion of Tregs and Tconvs led to a higher percentage of donor-reactive Tregs in both the spleen and graft (85). Treg accumulation in tolerant grafts and draining lymph nodes (dLNs) was also observed in αCD154+ rapamycin-treated mice that received rat islet xenografts (86). Corneal allograft recipient mice treated with αCD154 also exhibited increased graft-infiltrating Tregs and increased Treg to Tconv ratio in the spleen (87). Treg expansion was also observed in both the graft and dLNs of mice with spontaneously tolerant kidney allografts at both days 7 and 14 post-transplantation, along with an increase in splenic Treg proportions at day 14, possibly indicating initial Treg expansion in the graft followed by migration to the spleen (88).

A high Treg:Tconv ratio has been shown to be important in the maintenance of allograft tolerance. Indeed, transferring high numbers of alloreactive Tconvs to tolerant cardiac allograft recipient mice broke tolerance whereas low numbers did not (89). Similarly, in tolerant mice, transferring low numbers of alloreactive Tconvs in combination with αPD-L1 and αCD25 to partially deplete Tregs caused a loss of tolerance, suggesting that overwhelming Tregs can break established tolerance (89). In CoB + DST-treated tolerant mice, there was an enrichment of Foxp3 expression as well as an increased Foxp3^+^ Treg frequency in the allograft compared to that in the periphery or to rejecting allografts and syngeneic grafts (90–92). However, during *Listeria monocytogenes* (Lm) infection-mediated rejection, the high percentage of Foxp3^+^ cells in the CD4^+^ T cell compartment in the graft was reduced (91, 92). After resolution of Lm infection, the frequency of Foxp3^+^ Tregs in the graft returned to higher levels, accompanying a return of tolerance (92). Furthermore, after the infection was cleared, accumulation of Tregs reoccurred in a second donor-matched allograft, but αCD25-mediated depletion of Tregs at the time of second transplantation prevented acceptance of this new graft, indicating a role for Tregs in the restoration of tolerance (91).

### 3.2. Treg recruitment to the graft

As described above, Treg proximity to target cells is required for suppression. Therefore, Tregs must be recruited to the allograft to successfully constrain the Tconv-mediated rejection of the graft. DCs, particularly plasmacytoid DCs (pDCs), play a major role in the recruitment of Tregs to the graft and the induction of pTregs in the graft. During tolerance, pDCs picked up alloantigen in a cardiac allograft, migrated to peripheral LNs where they were necessary for the induction of Tregs and for tolerance (93). It has also been observed in mice that DBA/2 kidneys transplanted into B6 hosts are spontaneously accepted, while the reverse combination (B6 kidney transplanted into DBA/2 host) leads to rejection. Immunohistochemistry of the spontaneously accepted allografts showed Foxp3^+^-rich aggregates as well as the presence of SiglecH^high^ pDCs. *In vitro*, pDCs from accepted kidneys induced more Foxp3 expression in T cells after co-culture than did syngeneic pDCs, and these Tregs were highly suppressive of sensitized T cells. Adoptive transfer of Tregs induced by culture with allogeneic pDCs was also capable of prolonging donor-matched cardiac allograft survival (94). Non-transplanted mice receiving allogeneic liver pDCs had increased percentage and counts of Tregs in the liver, spleen, and LNs. Furthermore, the presence of pDCs in the graft promoted Treg accumulation, protecting against graft injury and rejection, and depletion of pDCs from donor livers prior to transplantation led to acute rejection of the graft (95). Thus, pDCs seem central to tolerance and upstream of Treg accumulation.

Additionally, CCR4-mediated Treg recruitment to the graft was required for costimulation blockade mediated tolerance induction (90). CCR4 expression is regulated in part by Foxp3 expression and has been shown to promote Treg infiltration in the tumor microenvironment (96). CCL22 is a ligand for CCR4 and is produced by DCs and macrophages, highlighting a possible mechanism for DC-mediated Treg recruitment to the graft (90, 97, 98). Another potential pathway through which crosstalk between pDCs and Tregs can promote Treg differentiation and accumulation in allografts is through expression IDO, which has been shown to promote differentiation of Tregs in models of autoimmunity (99–101). The interaction between CTLA-4 on Tregs and CD80/CD86 on DCs in turn promotes IDO expression by DCs (47). Soluble CTLA-4 in the form of the fusion protein CTLA4-Ig has similarly been shown to induce long term graft survival in cardiac allograft recipient mice in an IDO-dependent manner (102–104). Patients who received CTLA4-Ig treatment after kidney transplantation had increased IDO^+^ cells in the periphery and increased Tregs in graft biopsies compared to cyclosporine-treated patients (105).

Although DCs have been shown to play a major role in Treg induction and trafficking to the graft, they are not the only innate immune cell type that can both promote rejection and be implicated in tolerance. For instance, monocytes, and neutrophils have also been shown to play a role in Treg recruitment. Recipient Gr-1^+^ innate immune cells were shown to play a role in promoting Treg trafficking to skin grafts in a human skin graft to mouse xenogeneic model using transfer of radiolabeled Tregs (106). Furthermore, macrophages may also play a role in Treg-mediated prolongation of graft survival; erythropoietin was capable of mediating prolongation of murine kidney allograft survival (BALB/c into B6) in a myeloid cell-dependent manner (107). Erythropoietin receptor signaling on myeloid cells drove a shift to a protective phenotype in macrophages, which in turn induced expansion of donor-reactive Tregs (107).

### 3.3. Phenotype of graft-infiltrating Tregs

Tregs that are recruited to the graft during transplantation tolerance exhibit a tolerogenic phenotype. Tregs accumulating in tolerant grafts expressed higher levels of *TGFβ*, *IL-10*, *Cxcr3*, and *Blimp1* transcripts compared to splenic Tregs from both transplanted and naïve mice (88), as well as increased expression of Neuropilin-1 (Nrp1) and CD73 compared to Tregs in rejecting grafts (85). The majority of Tregs in tolerant grafts were PD-1^+^, but upon Lm infection the percentage of PD-1^+^ Tregs was reduced, which may account for part of the eroded tolerance observed after clearance of Lm infection (92). Similarly, liver Tregs induced by portal vein injection of allogeneic pDCs exhibited increased PD-1, Tim3, CD40l, and CD69 expression (95).

Inflammation due to tissue damage or infection poses a danger to graft survival due to the destabilizing effects of inflammation on Tregs. In a corneal transplant model, adoptively transferred Nrp1^−^ pTregs were susceptible to conversion to IL-17- and IFNγ-producing exTregs in dry eye disease hosts but not control hosts. Furthermore, corneal transplant hosts with dry eye disease exhibited a decreased frequency of Tregs in the dLNs and impaired Treg suppressive capacity (108). Endothelial cells exposed to inflammatory conditions upregulated ICAM-1, PD-L1 and exposure to these endothelial cells led to an inhibition of Treg differentiation (109). Since some infections are capable of precipitating rejection of previously stably accepted allografts (110–112), one potential mechanism for this may be the destabilization of Tregs due to the inflammatory response to infection. Better understanding of the stability of Tregs after transplantation, at steady state and after infections would help improve clinical outcomes and offers new avenues for research.

## 4. Tregs as a prognostic tool in human transplantation

The different fate of Tregs in tolerance and rejection has prompted for the frequency and phenotype of Tregs in human transplant recipients to be used as a prognostic tool. In kidney allograft recipients, early after transplantation (1 and 3 months post-transplantation), the number of circulating Tregs and Foxp3 expression were associated with improved allograft survival and function both at the time of sampling and over the course of 5 years (113). In clinical trials to induce transient mixed chimerism and sustained graft acceptance in recipients of combined kidney and bone marrow transplants from haploidentical donors, recipients who remained tolerant to their graft as long as 5 years after immunosuppression cessation exhibited an expansion of pre-existing donor-specific Tregs at 6 months post-transplantation compared to pre-transplantation, whereas this same population was reduced at the same time point in a recipient that was not tolerant (114, 115). These Tregs expressed higher levels of Foxp3 and performed better in *in vitro* suppression assays after restimulation with donor B cells, compared to restimulation with 3rd party B cells (115). In transplant patients, Tregs in peripheral blood had increased surface and intracellular Notch-1 expression compared to Tconvs (116). Despite this, Notch-1^low^ Tregs from human peripheral blood were more suppressive than Notch-1^high^ Tregs, revealing an avenue of further research to understand the role of Notch-1 expression and signaling in Tregs (116). In kidney allograft recipients, a higher level of Foxp3 mRNA in cells from urine samples not only correlated with better graft function, but Foxp3 mRNA levels also predicted the reversibility of a T cell-mediated rejection event (117, 118). This is particularly promising as it demonstrates non-invasive methods of harnessing the prognostic potential of Tregs in transplantation success.

## 5. Interventions that modulate Treg suppressive ability

### 5.1. Post-transcriptional modifications

There are diverse factors that can impact the suppressive ability of Tregs in a transplantation context, one of which is post-transcriptional modifications that affect Treg differentiation, function, and stability. For instance, N6-methyladenosine mRNA modification was involved in Treg differentiation and function, and was regulated by “reader” and “writer” proteins including methyltransferase like 14 (METTL14), Wilms tumor 1-associating protein (WTAP), and methyltransferase like 3, all of which were necessary for Treg function (119–122). The ability of these proteins to modulate Treg functional ability is important in tolerance. Mice with METTL14-deficient Tregs rapidly rejected allografts and had lower levels of IL-10 and TGFβ (121). Similarly, expression of WTAP in peripheral blood samples from tolerant kidney allograft recipients was correlated with a higher Treg percentage in the peripheral blood and WTAP overexpression in naïve CD4^+^ T cells resulted in Foxo1 upregulation, promoting Treg differentiation and suppressive ability as well as improving allograft survival in mice (122). Tregs expressed increased miR-146a, a microRNA that targets TRAF6 and IRAK1 to negatively regulate NF-κB activation during rejection, and miR-146a-deficient Tregs had improved expansion but impaired function, although IFNγ blockade in miR-146a-deficient mice rescued Treg function and led to prolonged allograft survival (123). Another important post-transcriptional modification impacting Tregs is the acetylation of Foxp3 downstream of IFNβ-mediated activation of pSTAT1 (34). This acetylation promotes Treg function and stability and promotes long term allograft survival (34, 124).

### 5.2. Metabolic changes

Cellular metabolism plays a significant role in T cell function, and there is an increasingly appreciated role for metabolic changes in the suppressive function of Tregs, particularly in the context of anti-tumor immunity and in lupus. For example, in a mouse model of lupus, excess iron promoted the differentiation of pathogenic T follicular helper (Tfh) cells and increased production of pro-inflammatory cytokines. Moreover, increased transferrin receptor (CD71) expression was correlated with worse disease outcome, whereas blocking CD71 led to increased Treg differentiation and IL-10 production (125, 126).

The effects of metabolic changes on Tregs have been especially characterized in tumor settings due to the numerous metabolic alterations in the tumor microenvironment. Tregs exhibit a different metabolic profile from Tconvs, and Treg function is reduced upon high glucose uptake but enhanced with lactic acid uptake and metabolism (127, 128). Fatty acid oxidation and oxidative phosphorylation pathways are highly active in suppressive proliferating Tregs (128). Reduction in Tconv glycolytic activity via mTOR inhibition results in a more Treg-like metabolic profile, and this mTOR inhibition-mediated Tconv functional shift also prevented the induction of graft-versus-host disease (GvHD) (129). The distinct metabolic profile of Tregs suggests a promising avenue of therapeutic targeting in transplantation, as modulating glycolysis, oxidative phosphorylation, and fatty acid oxidation pathways could promote Treg proliferation and suppression without triggering Tconv activation.

### 5.3. Therapeutic interventions that affect Treg function

**Figure 2:**
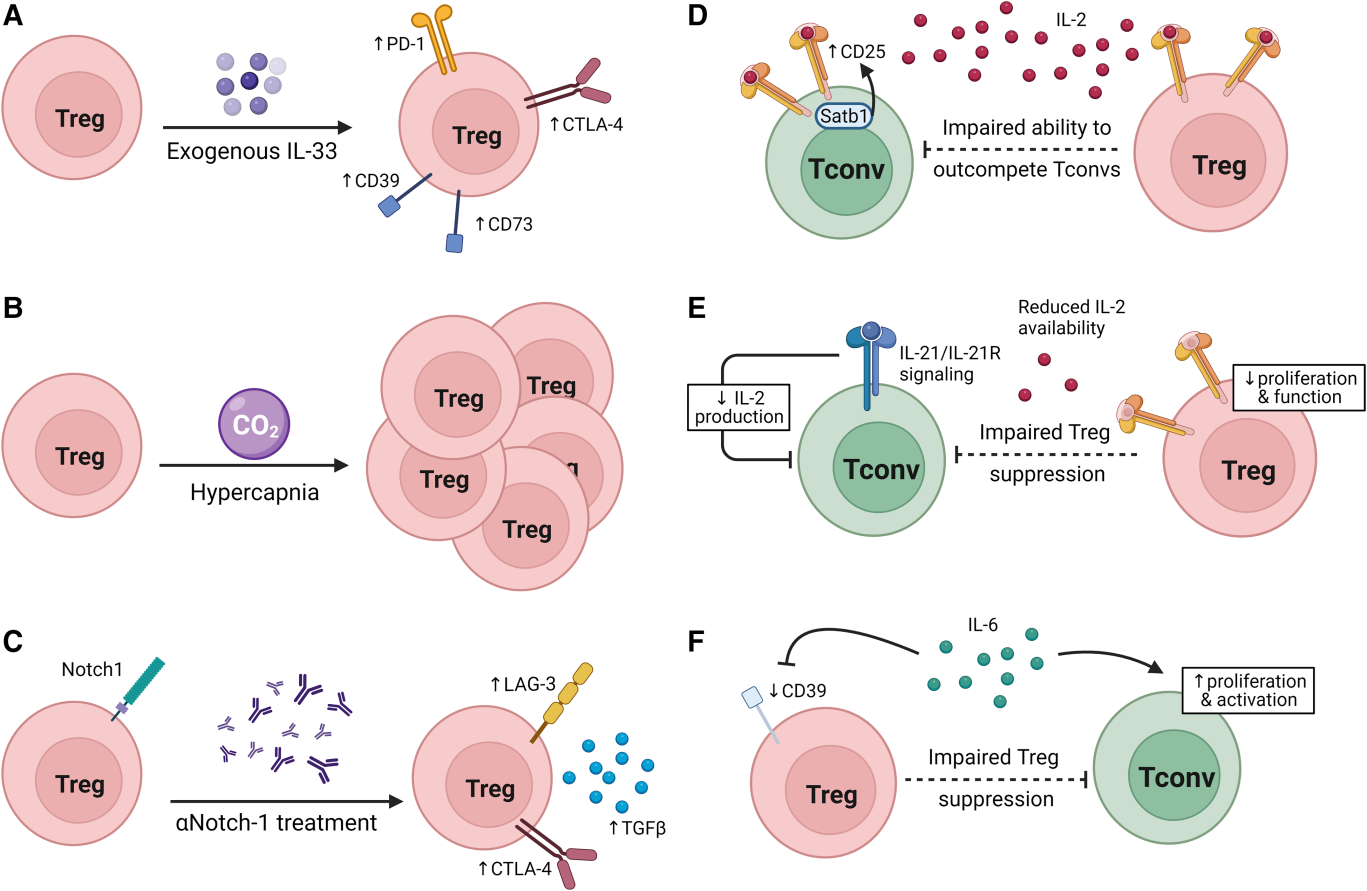
Modulating Treg suppressive ability. (**A–C**) Interventions that modulate Treg suppressive capacity in transplantation. (**A**) IL-33 treatment increases inhibitory receptor and CD39/CD73 expression on Tregs. (**B**) Hypercapnia promotes Treg expansion. (**C**) Neutralization of Notch-1 signaling increases inhibitory receptor expression and TGFβ production. (**D–F**) Factors that alter the susceptibility of Tconvs to Treg suppression. (**D**) Satb1 expression promotes CD25 expression on Tconvs, reducing Tregs’ ability to outcompete Tconvs for IL-2. (**E**) IL-21 signaling in Tconvs reduces IL-2 production, impairing the ability of Tregs to suppress Tconvs without impeding Tconv survival and function. (**F**) IL-6 signaling on Tregs can drive a reduction in CD39 expression by Tregs while IL-6 signaling drives Tconv activation and proliferation.

Treg phenotype and suppressive ability can also be affected via different therapeutic interventions (Figures 2A–C). In addition to inhibition the proliferation of Tconvs, CoB (αCD154 treatment) in transplant hosts was shown to augment Treg suppressive ability (130). In diabetic B6 Foxp3^DTR^ mice transplanted with BALB/c islets, after normoglycemia was achieved with αCD154 treatment, Tregs from αCD154-treated tolerant mice suppressed B6 Tconvs better than Tregs from naïve mice when cultured with donor-matched BALB/c splenocytes compared to culture with third-party C3H splenocytes, indicating a donor-specific effect (131).

In line with the observation in both mice and humans that Notch-1^low^ Tregs were more suppressive than Notch-1^high^ Tregs in transplant recipients, Notch-1 blockade treatment prolonged allograft survival in a Treg-dependent manner (116). Furthermore, αNotch-1-treated cardiac allograft recipient mice not only had an increased proportion of Tregs in the spleen and graft, but splenic Tregs from these mice also expressed more LAG3, CTLA-4, Ki67, produced more TGFβ, and performed better in an *in vitro* Tconv suppression assay. Notch-1-deficient (N1c-KO) Tregs also outperformed wildtype (WT) Tregs in an *in vitro* suppression assay, and transfer of N1c-KO Tregs and WT CD4^+^ Tconvs improved graft survival in RAG-KO allograft recipients compared to WT CD4^+^ Tconvs and Tregs. Notch-1 inhibition in a humanized skin graft model reduced overall T cell infiltration to the graft, and increased the proportion of Tregs in the graft (116). These data emphasize the promise of using clinical observations of Treg phenotype post-transplantation to reveal targetable mechanisms to promote tolerance.

It has been shown in cancer, allergy, and lung injury that IL-33, an alarmin expressed at higher levels during inflammation (132, 133), promotes tissue repair and a more suppressive phenotype in ST2^+^ Tregs (134–138). However, IL-33 in the contexts of transplantation and GVHD has pleiotropic effects; there is evidence of IL-33 promoting Treg expansion, but IL-33 also has been shown to act as a costimulatory molecule for alloreactive Tconvs (138–142). In a GVHD model, IL-33/ST2 signaling in CD4^+^ cells promoted expansion and led to upregulation of Tbet while inhibiting IL-10 and Foxp3 expression which led to an exacerbation of GVHD severity (142, 143). Conversely, there is also evidence of IL-33 promoting ST2^+^ Treg expansion and therefore having a protective effect against GVHD (139). For instance, in a murine cardiac allograft model, IL-33 treatment not only prolonged graft survival but induced Foxp3^+^ Treg expansion in both the graft and spleen (140, 144). Treatment with exogenous IL-33 in skin allograft recipient mice caused a transcriptomic skewing towards an anti-inflammatory/tolerogenic phenotype, leading to an expansion of Foxp3^+^ cells in spleen, LNs, and periphery within the CD4^+^ population. These Tregs expanded after IL-33 treatment and expressed higher levels of the co-inhibitory receptors CTLA-4 and PD-1 as well as of CD39 and CD73 which are both involved in adenosine metabolism. Furthermore, IL-33 treatment led to long term graft survival in a skin allograft model (145). The complexities of the impacts of IL-33 on both Tregs and Tconvs in transplantation tolerance and rejection are yet to be fully understood.

Intermittent exposure to CO_2_ leads to hypercapnia, and subsequent prolongation of skin allograft survival. Mice with hypercapnia exhibited an increased percentage of Tregs in the CD4^+^ T cell compartment of both LNs and spleen as well as significantly decreased serum levels of pro-inflammatory cytokines IFNγ, TNFα, and IL-6. This may be due to increased numbers of iTregs since intermittent CO_2_ exposure-treated naïve T cells differentiated into Tregs at a higher rate than untreated cells (146). Interestingly, in the tumor environment, hypoxia has been shown to lead to increased extracellular adenosine and increased cAMP through adenosine receptor signaling, potentially indicating a mechanism through which hypercapnia may allow Tregs to suppress Tconvs and prolong graft survival (147, 148).

In a corneal allograft model, treatment with TIGIT-Fc, which significantly increased the mean graft survival time, led to increased Helios^+^ cells and also increased Foxp3^+^ cells compared to the PBS-treated control group. TIGIT-Fc treatment led to better suppression in a Treg suppression assay. The increased number and suppressive ability of Tregs after TIGIT-Fc treatment correlated with better graft survival (149).

Thus, several interventions can modulate Treg suppressive function *in vivo*, contributing to improved allograft survival. Whether some of these interventions can be combined or whether the maximal donor-specific suppression by Tregs is achieved by a single treatment remains to be investigated.

### 5.4. Clinical interventions that affect Treg function

Clinical treatment regimens for transplant recipients can be broadly divided into induction therapies and maintenance therapies. Induction therapies such as basiliximab and anti-thymocyte globulin (ATG) are often used in combination with longer term maintenance immunosuppression. The current standards of care for maintenance immunosuppression can be grouped into calcineurin inhibitors (CNI) such as cyclosporine and tacrolimus, mTOR inhibitors such as rapamycin/sirolimus, anti-proliferative therapies such as mycophenolate mofetil (MMF), JAK inhibitors, and co-stimulation blockade therapies such as belatacept. Unfortunately, many of these treatments are limited due to their depleting, destabilizing or otherwise inhibiting effects on Tregs (150–154).

Induction therapies are important for preventing acute rejection soon after transplantation (155). Rabbit anti-thymocyte globulin (rATG) is used to deplete lymphocytes and, along with basiliximab, a non-depleting IL-2R agonist, are commonly used induction agents (155). Basiliximab has been shown to lead to a decrease not only in Treg frequency, but also functional capacity. For instance, *in vitro*, basiliximab added to T cell culture from healthy donors led to a significant decrease in Treg frequency but not in the overall CD4^+^ or CD8^+^ T cell populations (156). Basiliximab suppressed Treg proliferation and IL-10 production *in vitro*, both functions which are known to be impacted by IL-2 signaling (156). Crucially, in a study of pediatric heart transplant recipients, the absolute number of CD4^+^Foxp3^+^CD25^+^ Tregs was significantly reduced compared to both pre-transplant levels and to patients who did not receive basiliximab (156). Kidney transplant recipients treated with basiliximab induction therapy had decreased circulating Tregs at both 1 and 2 weeks post-transplantation compared to patients who did not receive any induction therapy (151). A significant reduction in circulating Tregs in kidney recipients treated with basiliximab in combination with CNI + MMF + steroid therapy compared to patients who received only triple immunosuppression has been observed as early as 1 day post-transplantation and as far as 6 weeks post-transplantation (157). Basiliximab treatment also decreased the expression of CD25 on Tregs (157). In contrast to the effects of basiliximab, lymphocyte depletion via rATG may allow for the expansion of functional Tregs. *In vitro*, human peripheral blood mononuclear cells (PBMCs) cultured with rATG exhibited a conversion of CD4^+^CD25^−^ T cells to CD4^+^CD25^+^ Tregs that were functionally suppressive (158). In both basiliximab and rATG induction therapies, Treg absolute number has been observed to decrease, but crucially the Treg frequency in rATG-treated recipients actually increased due to the decrease in Tconvs also observed in rATG induction therapy (154, 159, 160). Furthermore, rATG treatment has been shown to promote expansion of peripheral Tregs which likely contributed to the faster kinetics of Treg reconstitution in rATG-treated patients over basiliximab-treated patients (160–162). In a comparative study of islet allograft transplant recipients receiving either αCD25 or rATG induction therapies, ATG recipients maintained a stable frequency of CD25^+^CD4^+^ T cells, whereas the frequency of these Tregs decreased significantly in αCD25 induction therapy recipients (163). Of note, both groups had similar rates of insulin independence 1 year after islet transplantation (163), though the functional status of allospecific Tconvs was not compared.

CNIs such as cyclosporine (CsA) and tacrolimus are widely utilized immunosuppressive therapies in transplant recipients. These treatments block TCR-mediated activation of T cells and also block activation-induced cell death of T cells, which impairs the ability of co-stimulation blockade to induce tolerance (164–168). Additionally, CNIs have been shown to reduce peripheral Treg frequency in kidney transplant recipients (150, 169) which could limit the efficacy of these treatments. Importantly, clinical data on the effect of CNIs on Tregs are not all in agreement; for instance, in a study of kidney transplant recipients using either rapamycin or CsA as maintenance immunosuppression, no difference in circulating or graft-infiltrating Tregs was observed (168).

Belatacept, a CTLA4-Ig treatment, inhibits CD28 interaction with CD80/CD86, which is a critical pathway not only for Tconv activation but also for Treg homeostasis (170). Wildtype B6 mice administered four doses of CTLA4-Ig had half of the total Tregs by both percentage and absolute number than did their untreated counterparts (171). However, the effects of CTLA4-Ig on Tregs can be mixed. Cardiac allograft recipient mice that received 2 doses of CTLA4-Ig exhibited graft-reactive splenic Treg expansion at similar levels as αCD154 + DST-treated mice, but recipients treated with twice weekly CTLA4-Ig for 4 weeks showed a decrease in the percent of donor-specific Tregs (85), suggesting that more transient blockade of CD28 may preserve Treg viability. Indeed, it has been shown that the graft-protective effects of CTLA4-Ig are dependent on Tregs only at low doses, but not at high doses (172). Furthermore, CTLA4-Ig administration in murine recipients of a fully mismatched cardiac allograft prolonged graft survival but treatment in recipients of an MHC-II-mismatched allograft precipitated rejection (171). This could be due to the number of alloreactive Tconvs being smaller in a partially mismatched model, whereas in a fully-mismatched model the higher number of alloreactive Tconvs allows for CTLA4-Ig to have a greater effect on Tconvs than Tregs (171). Since CTLA4-Ig treatment may have negative impacts on Tregs, it may be beneficial to combine costimulation blockade therapies with Treg expanding interventions. For instance, IFNβ, which has been shown to promote Treg induction, synergized with CTLA4-Ig to prolong cardiac allograft survival in a murine model (34). Similarly, the addition of IL-2/αIL-2 complexes to CTLA4-Ig treatment in a murine cardiac allograft model reversed the observed decrease in Treg frequency observed with CTLA4-Ig and prolonged graft survival over CTLA4-Ig alone (173). These data illustrate the potential promise of combining Treg promoting therapies with more traditional immunosuppression. Belatacept has been approved for use in kidney transplant recipients and the BENEFIT Phase III study compared two belatacept regimens with CsA treatment (174). Interestingly, although belatacept treatment was associated with an increase in acute rejection episodes, by 1 year post-transplantation belatacept-treated recipients had similar patient and graft survival as CsA-treated recipients and by 7 years post-transplantation, belatacept-treated recipients had higher patient and graft survival as well as graft function than did CsA-treated patients (174, 175). Of note, in mixed lymphocyte reactions using Tregs from healthy volunteers, belatacept treatment inhibited both proliferation and Treg generation (153) highlighting the potential drawbacks of belatacept as a treatment option.

Inhibiting the JAK/STAT pathway is also a target pathway of interest to develop treatment regimens without the use of CNIs. A JAK3 inhibitor, CP-690,550, was efficacious in prolonging graft survival in mouse and monkey transplant recipients (176, 177). Alloactivated Tconvs from the peripheral blood of healthy individuals exhibited impaired proliferation when treated with CP-690,550 both in the presence and absence of Tregs (178). Furthermore, Tregs from human kidney transplant recipients treatment with CP-690,550 maintained functional suppressive abilities (178, 179). Despite the preservation of Treg functional capacity, CP-690,550 treatment led to a decrease in the percent of CD4^+^CD25^high^ T cells from their pre-treatment baseline (179).

Unlike the therapies discussed above, mTOR inhibitors such as rapamycin (also known as sirolimus) may in fact be permissive of Treg function, expansion, and survival. Rapamycin binds to the FK506-binding protein-12 (FKBP12) but, unlike CsA or tacrolimus, rapamycin does not act as a CNI but rather inhibits mTOR (165, 168, 180, 181). Thus, rather than blocking TCR-mediated activation, rapamycin blocks CD4^+^ T cell progression through the cell cycle (165, 181). *In vitro* evidence has shown that rapamycin exposure leads to an expansion of functionally suppressive CD25^hi^CD4^+^ T cells, which are capable of preventing islet allograft rejection when adoptively transferred into recipient mice (165), and are more suppressive than Tregs cultured without rapamycin (182). Furthermore, rapamycin treatment in rats resulted in an increased ratio of CD25^+^CD4^+^ to CD25^−^CD4^+^ T cells (181). Rapamycin treatment also promoted the stability of phenotypic stability in an inflammatory context (183). In clinical contexts, rapamycin treatment in kidney transplant recipients did not cause a reduction in circulating Tregs (150, 169). Additionally, when human CD4^+^CD25^+^ Tregs were cultured with rapamycin, they outperformed Tregs cultured with CsA in an *in vitro* suppression assay, and more importantly were able to suppress on-going T cell responses in a mixed lymphocyte reaction (184). The benefits of rapamycin can also be observed in combination with other therapies. In a Phase II study, kidney recipients receiving a combination of rATG, belatacept, and sirolimus (rapamycin) not only had no incidence of acute rejection at 1 year post transplantation, but also had significantly higher frequencies of Tregs in the periphery than patients treated with combination therapies of rATG/belatacept/MMF, rATG/tacrolimus/MMF, or basiliximab/belatacept/MMF/steroids (185). Tregs from patients treated with rATG/belatacept/sirolimus also were highly suppressive in an *in vitro* suppression assay (185). Kidney transplant recipients treated with rapamycin did not experience a decrease in peripheral or kidney-infiltrating Treg frequencies (168).

It is important to note that existing research on the effects of post-transplantation therapies on Tregs, and the impact of potentially inhibited Tregs on graft survival and function are not yet fully understood. A more complete understanding of the impact of commonly used post-transplantation therapies is necessary for creating treatment regimens that preserve or promote Treg frequency and functionality. Accounting for Treg survival in post-transplantation treatment could be beneficial in promoting long-term graft survival and function, especially with the eventual goal of immunosuppression-free donor-specific tolerance.

### 5.5. Susceptibility of Tconvs to suppression and Tconv hypofunction

It is also conceivable that Tregs play a role in programming the Tconv hyporesponsiveness to alloantigen that can occur following tolerance induction (186), although this would need to be formally tested. In the tumor microenvironment, IL-10-producing and IL-35-producing Tregs together drive Blimp1 expression and an exhausted phenotype in CD8^+^ tumor-infiltrating T cells (187). In the transplantation setting, there is a correlation between hyporesponsiveness of alloreactive T cells and the presence of Tregs. For instance, in renal transplant patients, impaired production of IFNγ by PBMCs in response to donor-specific stimulation was associated with increased numbers of Tregs (188). An association between the presence of Tregs and more exhausted alloreactive T cells was also observed in a liver transplant model. Mice given pDC-depleted liver allografts not only had significantly decreased numbers and percentages of Tregs in both the graft and LNs, but also had fewer graft-infiltrating CD4^+^ and CD8^+^ T cells expressing the exhaustion markers Tim-3, PD-1, and CTLA-4. There were also increased levels of granzyme B, perforin and PD-L1 in pDC-depleted grafts, and CD4^+^ T cells from these livers produced significantly higher levels of IL-2 and TNFα. CD4^+^ and CD8^+^ T cells from recipients of pDC-depleted allografts had increased proliferation and *in vitro* pro-inflammatory cytokine production (IL-6, TNFα, IFNγ) compared to T cells from pDC-replete graft recipients (95). Whether this association between Tregs, pDCs and hyporesponsive or exhausted alloreactive T cells in transplantation tolerance is causal remains to be addressed.

Transplantation tolerance is not solely reliant on the suppressive ability of Tregs but also on the susceptibility of Tconvs to Treg suppression (Figures 2D–F). We reported that Tconvs deficient for the transcription factor Satb1 had impaired ability to upregulate CD25 upon TCR stimulation, allowing Tregs to outcompete them for IL-2 and leaving Satb1-deficient Tconvs more susceptible than control Tconvs to Treg suppression. Moreover, in a murine cardiac allograft model, mice with Satb1-deficient Tconvs and WT Tregs had significantly improved graft survival than mice with WT Tconvs and Tregs (189). Conversely, IL-21 inhibits the ability of Tregs to suppress Tconvs along the same axis. Indeed, IL-21/IL-21R signaling on Tconvs inhibited IL-2 production by Tconvs, impairing Treg proliferation and homeostasis while leaving Tconvs largely unaffected due to the ability of IL-21 to act in a similar manner as IL-2 on Tconvs (190, 191). However, the impact of IL-21 on the ability of Tregs to suppress Tconvs remains to be shown in transplantation settings.

Another axis through which Treg suppressive ability may be modulated in transplantation tolerance is IL-6 and TNFα signaling through PKB/c-Akt. It has been shown in multiple sclerosis, rheumatoid arthritis, psoriasis, and juvenile idiopathic arthritis that IL-6 and TNFα activate PKB/c-Akt in Tconvs and impair Treg suppression of Tconvs, permitting Tconv proliferation and activation. IL-6 receptor blockade or PKB inhibition both restored the suppressive capacity of Tregs (32, 192–198). Mechanistically, IL-6 may impair Treg suppression as a consequence of downregulating CD39 expression by Tregs (32, 192–198). This pathway has also been implicated in transplantation. IL-6 deficiency or its blockade in mouse models and human allograft recipients was accompanied by Treg expansion and prolonged graft survival (199–203). Improved suppressive capacity has also been observed in Tregs from IL-6-deficient allograft recipients, though the mechanism for this has not yet been defined (201). Since IL-6 is produced by DCs in response to damage associated with transplantation and in response to antigens (199, 204, 205), further understanding of the mechanisms conferring Tconv resistance to Treg suppression in transplantation is warranted.

## 6. Therapeutic potential of Tregs

### 6.1. Preclinical data

#### 6.1.1. Donor-specific Tregs

Given the association between Tregs and better graft outcomes or responsiveness to immunosuppressive therapies, Tregs have been considered as a possible cell therapy to improve clinical transplant fate. One consideration for therapeutic harnessing of Tregs in clinical settings is choosing which Tregs to use. Polyclonal Tregs are more easily expanded or isolated, but donor-specific Tregs are likely more effective. In a murine islet allograft model, after recipient pre-conditioning by T cell depletion, transfer of fewer donor-reactive than polyclonal Tregs achieved indefinite graft survival (206). Transfer of iTregs stimulated *in vitro* with alloantigen prevented both acute and chronic rejection in a mouse model of bone marrow transplantation and cardiac or skin transplantation (207). Additionally, Tregs retrovirally transduced to express TCRs specific for donor peptide presented on host MHC-II were able to induce long-term allograft tolerance and protect against cellular infiltration of heart allograft (208). *In vitro* expanded donor-specific Tregs were functionally suppressive *in vivo* and trafficked to allografts where they were able to induce donor-specific tolerance better than polyclonal Tregs (82, 209, 210). The ability of alloreactive iTregs to promote donor-specific tolerance was augmented by the generation of these iTregs in the presence of vitamin C, which promoted lineage stability (211). This is an important finding since one major drawback of *in vitro* expanded Tregs is the potential for a loss of Foxp3 expression and Treg functionality (212).

Mouse data indicating more protection from donor-specific Tregs have been recapitulated with human cells. Human Tregs with direct allospecificity could be enriched and expanded and were more protective in a human skin xenotransplantation model (213). Furthermore, purified, expanded alloantigen-specific human Tregs more potently inhibited donor-reactive Tconvs *in vitro* than did expanded polyclonal Tregs (214). The findings that antigen-specific Tregs are more effective in tolerance induction are a further reason to pursue chimeric antigen receptor (CAR)-Treg therapies in which the antigen specificity of the Tregs can be manipulated.

#### 6.1.2. Expansion of endogenous Tregs

As an alternative to transferring Tregs expanded *in vitro*, some groups have focused on expanding endogenous Tregs *in vivo*, using various approaches to deliver IL-2. To prevent low dose IL-2 treatment from expanding Tconvs, engineering approaches have been attempted. In a cardiac allograft model, transfer of Tregs expressing an engineered IL-2 receptor that binds exclusively to an engineered version of IL-2 led to indefinite heart graft survival after treatment with tacrolimus and engineered IL-2, and survival persisted after cessation of tacrolimus treatment (215). Other approaches to IL-2-mediated expansion of endogenous Tregs are also of interest. A controlled release microparticle system delivering TGFβ, IL-2, and rapamycin led to donor-specific tolerance characterized by indefinite graft survival, spontaneous acceptance of a second graft but rejection of a third-party graft, in a rat hindlimb major mismatched model. This system led to an increase in Treg frequency and a decrease in IFNγ^+^ Th1 cells when compared to untreated rats. Moreover, these Tregs were more effective at suppressing donor-reactive Tconvs than those from untreated rats (216). In a murine cardiac allograft model, low dose IL-2 administration, in combination with co-stimulation blockade, increased the frequency of Tregs in the graft, protecting against fibrosis and prolonging graft survival (217). Treatment of cardiac allograft recipient mice with IL-2/anti-IL-2 complexes in combination with CTLA4-Ig rescued the decrease in Tregs that usually accompanies CTLA4-Ig treatment because of its ability to block CD28 signals that are important for Treg survival, and significantly prolonged graft survival and health (173).

#### 6.1.3. Donor-derived Tregs

It is well appreciated that recipient Tregs play an integral role in transplantation tolerance. However, the contribution of donor-derived Tregs, either transferred as passenger cells within the graft or adoptively transferred as therapy, is less well understood. Adoptive transfer of donor-derived nTregs prolonged murine cardiac allograft survival more effectively than recipient Tregs (218). Donor Tregs present in the graft itself were protective as evidenced by Treg depletion from the graft peri- and post-transplantation resulting in much faster rejection and more autoantibody response (218). Allogeneic donor-derived Tregs were able to inhibit *in vitro* IFNγ production by both CD8^+^ and CD4^+^ T cells more effectively than syngeneic or 3rd party Tregs. This inhibition was TGFβ- and IL-10-dependent and could be reversed by addition of IL-2 to the culture. Furthermore, *in vivo* adoptive transfer of donor-derived Tregs prolonged skin allograft survival and induced donor-specific tolerance (219).

#### 6.1.4. Treg allorecognition pathways

An important consideration in the use of transferred alloreactive Tregs to promote transplant survival is whether they should recognize alloantigen directly or indirectly. Direct allorecognition refers to a T cell recognizing donor MHC expressed on donor parenchymal cells of the graft or on donor APCs (220). Conversely indirect allorecognition occurs when a T cell recognizes alloantigen that has been processed and presented by host APCs (peptides from donor MHC or from any protein with polymorphisms distinct between the donor and host) (220). Tconvs activated by direct allorecognition contribute more to early acute graft rejection since donor APCs are short lived, whereas effector T cells with indirect allospecificity have been shown to drive both acute and chronic rejection (220). Whether it would be more desirable to use direct or indirect Tregs depends on their specificity and mechanism of Tconv suppression. Direct allorecognition for antigens expressed constitutively on the graft, such as donor MHC Class I, would allow Tregs to localize to the graft after their transfer, whether they are transferred early or late after transplantation. In contrast, direct allorecognition for alloantigens expressed more transiently in the graft, such as donor MHC Class II, may not be as useful if MHC Class II-expressing donor APCs migrate out of the graft or die shortly after transplantation. If a major mechanism of Treg suppression is bystander, via secretion of suppressor cytokines or engagement of co-inhibitors on Tconvs such that they can suppress all Tconvs that reach the graft, then direct specificity for constitutively expressed MHC Class I may be optimal. A potential problem in sensitized hosts is if the target of Treg recognition (say donor MHC Class I) is also the target of existing alloantibodies that may coat the protein and prevent Treg access and Treg activation. If a major mechanism of Treg suppression is via inactivating APCs that are also presenting antigens to Tconvs, then indirect Tregs may be optimal, especially for peptides derived from donor MHC Class I that may be presented by host APCs throughout the life of the graft. For *ex vivo* generation of Tregs, stimulation with donor APCs expands Tregs with direct allospecificity, whereas Tregs with indirect allospecificity can be expanded *in vitro* by culture with either APCs that co-express host MHC Class II and donor MHC such that host MHC Class II can present peptides from donor MHC, or with host APCs pulsed with alloantigen (207, 221, 222). It has been demonstrated in murine models of both skin and cardiac allografts that while Tregs with direct allospecificity, expanded *ex vivo* with donor APCs are sufficient to prevent acute graft rejection, they are not enough to prevent chronic rejection from occurring (207, 208, 222). Adoptive transfer of Tregs that recognize alloantigen through both the direct and indirect antigen recognition pathways expanded *ex vivo* with F1 APCs, however, are capable of also efficiently inhibiting chronic graft rejection in an antigen-dependent manner (207, 208, 222). This is an important consideration when weighing methods of expanding Tregs for adoptive transfer therapies in the clinic since it seems that Tregs with indirect allospecificity may be better poised to promote long-term graft survival.

#### 6.1.5. CAR-Tregs

There is growing interest in the use of CAR-Tregs, Tregs engineered to express an extracellular binding domain (most often an antibody variable domain or a TCR), frequently specific for a donor-expressed HLA class I, since these molecules are only expressed in the graft and remain constitutively expressed. CAR-Tregs bypass concerns about limited precursor frequency of endogenous donor-specific Tregs.

Since mismatched HLA is an appealing target for CAR-Treg therapies, multiple groups have designed CAR Tregs with a surface antibody directed to HLA-A2 (A2-CAR). Infusion of A2-CAR Tregs to mice transplanted with a cardiac allograft transgenic for HLA-A2 improved graft survival over that in untreated mice and in polyclonal Treg-infused recipients and A2-CAR Treg infusion in combination with rapamycin treatment improved survival over rapamycin alone (223). The efficacy of donor-specific CAR Tregs has also been demonstrated in unsensitized skin allografted mice, leading to prolonged graft survival and decreased humoral response (224). However, this effect was not observed in previously sensitized mice, indicating that sensitization-induced alloantibodies may mask the target of CAR-Tregs and reduce the efficacy of this treatment (224). The use of these A2-CAR Tregs has been shown to be effective in skin xenograft and humanized mouse models, in which they suppressed alloreactivity and prolonged graft survival (225–228). A2-CAR Tregs demonstrated improved suppressive abilities over both polyclonal Tregs and Tregs bearing an irrelevant CAR, demonstrating the promise of this therapy (223, 225–228). However, although donor-reactive CAR Tregs have shown an ability to improve allograft survival, few CAR Tregs were found at later time points in a murine model, showing a lack of CAR-Treg persistence and a gradual loss of immunosuppressive control (223). Additionally, persistent signaling in CAR-Tregs recognizing ubiquitously expressed antigens, such as HLA Class I antigens, appeared to cause an exhausted phenotype and impaired suppressive functionality (229, 230). This could present a significant issue for the use of CAR-Tregs in transplantation since the currently developed A2-CAR Tregs would persistently engage their activating antigen. However, culture with rapamycin and vitamin C has been shown to restore CAR-Treg function after tonic signaling-induced dysfunction (230). These findings, in conjunction with the effects of mTOR inhibition described above, reveal a potential method of addressing the shortcomings of CAR-Treg therapy while avoiding adverse effects on endogenous Tregs.

### 6.2. Clinical data

There have also been attempts at expanding endogenous recipient Tregs *ex vivo* and reinfuse them for therapeutic benefit. For instance, human Tregs have been successfully purified from peripheral blood and expanded *ex vivo* then reinfused, showing feasibility of this approach, although more work on efficacy is necessary (231, 232). In human liver allograft recipients, Tregs from liver perfusate were capable of suppressing proliferation and IFNγ production by both donor and recipient T cells, and early after transplantation donor liver-derived Tregs could be found in the peripheral blood of patients, suggesting that early suppression of direct allorecognition response via donor Tregs may be useful to promote tolerance (233).

The multi-center Phase I/IIA ONE study is the largest clinical trial investigating the use of Tregs for the induction of transplantation tolerance to-date (234–236). The ONE study investigated several types of cell therapies, testing the efficacy and safety of polyclonal Tregs, donor-reactive Tregs, tolerogenic DCs, and regulatory macrophages (235–237). As the effects of tolerogenic DCs and macrophages is outside the scope of this review, we will focus on describing the results as they pertain to Treg cell therapy compared to conventional immunosuppressive treatments. Importantly, the ONE study found that the use of immune cell therapy for transplant recipients was overall both safe and feasible (235), which opens the door for future studies of these therapies. Transferred autologous Tregs were either expanded *ex vivo* with donor antigen (donor-antigen reactive Tregs, darTregs) or with TCR stimulation and co-stimulation (polyclonal Tregs) (235, 236). Monitoring of cell therapy recipients showed that even in darTreg recipients, there was not a loss of Foxp3 demethylated regions specific to Tregs which indicated Treg phenotypic stability, a crucial finding for the use of Tregs as a therapy (235). Despite the exciting findings of this study, further research is needed to fully investigate the efficacy of Treg transfer therapies. The ONE study is currently being followed up with a Phase IIB study termed the TWO study aiming to determine the efficacy of using *ex vivo* expanded autologous Tregs to minimize the need for immunosuppression (238, 239).

Although most CAR-Treg data have been confined to murine studies, and findings on the use of CAR-Tregs in the clinic have yet to be published, the promise of CAR-Tregs is being evaluated in active clinical trials. The STEADFAST study is an active Phase I/II clinical trial assessing the safety and efficacy of autologous HLA-A2 CAR-Tregs in kidney transplant recipients (240). Similarly, the LIBERATE study is an active Phase I/II clinical trial investigating the use of autologous HLA-A2 CAR-Tregs in liver transplant recipients (241). These active trials highlight the importance of continuing basic research for a more complete understanding of the role of Tregs in transplant tolerance and in Tconv hypofunction. Additionally, although HLA-A2 mismatch is an appealing target due to its prevalence, identifying CAR targets other than HLA-A2 should be pursued for use in patients with HLA-A2-negative grafts.

## 7. Discussion and limitations

Tregs play a crucial role in transplantation tolerance due to their ability both to suppress Tconvs which are known drivers of transplant rejection, and to drive a more tolerogenic milieu. Tregs suppress Tconvs through direct cell-cell contact with either T cells or APCs, as well as through contact-independent mechanisms that alter the environment. After transplantation, Tregs migrate to and accumulate in the allograft, as they are recruited by DCs. Surprisingly, Tregs accumulate in similar numbers in both tolerant and rejecting allografts, but the ratio of Tregs:Tconvs is significantly higher in tolerant grafts due to the lack of Tconv expansion in tolerance compared to rejection. Despite the comparable accumulation in the graft, Tregs from tolerant allografts exhibit a more tolerogenic phenotype, with increased expression of co-inhibitory receptors and anti-inflammatory cytokines. These observations translate to the clinical setting, where Tregs can be used as a prognostic tool. Higher frequencies of Tregs have been correlated with improved graft function and survival and better responsiveness to immunosuppressive therapy in cases of rejection episodes (113, 115, 117, 118). However, the ability of human Tconvs to transiently upregulate Foxp3 during activation without acquiring a regulatory phenotype (242–245), a phenomenon that does not occur in mouse Tconvs, can blur phenotypic distinctions between Tconvs and Tregs such that some of these human studies should be interpreted cautiously. Although the mechanisms of Treg suppression have been well studied, the factors that can challenge or augment Treg stability and suppressive ability in humans could still be better understood, to allow development of therapies that can prevent rejection and promote the suppressive ability of Tregs to enable long-term allograft acceptance or even donor-specific tolerance.

The promise of targeting Tregs to promote long term allograft stability has generated interest in using Tregs in clinical settings. However, there are currently multiple limitations constraining the therapeutic use of Tregs. Expansion of endogenous Tregs via IL-2 administration carries the risk of expanding alloreactive Tconvs that upregulate CD25 upon alloantigen recognition and needs therefore to be performed carefully to only target Tregs. This concern is highlighted by the results of a recent clinical trial in which low dose IL-2 treatment not only induced an expansion of circulating Tregs but also precipitated an IFNγ-driven inflammatory response that primed liver allografts for rejection (246). Furthermore, although circulating polyclonal Tregs were expanded by low dose IL-2 treatment, neither circulating donor-specific nor graft Tregs were preferentially expanded (246). The lack of targeted donor-specific Treg expansion in response to currently available IL-2 treatments is a major limitation in the feasibility of these interventions. However, there are exciting steps being taken to address the drawbacks of IL-2-mediated Treg expansion. Engineered “ortho IL-2” which activates only an engineered IL-2 receptor transduced to be present on Tregs has been shown in murine models to induce Treg expansion without impacting Tconvs (215, 247). The use of this ortho IL-2/IL-2R system also promoted donor-specific tolerance in a murine cardiac allograft model (215, 247). In these studies, allografts were tolerated even after tacrolimus treatment was stopped (215) which indicates a promising avenue of harnessing Tregs to induce lasting donor-specific tolerance without relying on lifelong immunosuppression.

The use of transferred Tregs in a therapeutic context is also limited by the potential loss of the Treg phenotype. This limitation impacts all types of Treg therapies. Expansion of Tregs by repeated *in vitro* stimulation has been shown to inhibit Treg stability and functionality (248, 249). Autologous donor-reactive Tregs adoptively transferred to lymphodepleted cardiac allografted cynomolgus monkeys were unable to prolong graft survival, and were found to have downregulated expression of Foxp3, Helios, CTLA-4, and Ki67 after transfusion despite *in vitro* demonstration of suppressive ability (250). This may be addressed by culturing Tregs for adoptive transfer in the presence of rapamycin which, along with minimizing the number of repeated stimulations, promotes Treg persistence and suppressive function (182). In humans, transferred Tregs exhibited a decrease in donor reactivity after transfusion (232). Genetic manipulation of Tregs may be an effective approach to promoting survival and stability in transferred Tregs. For instance, forced expression of Foxp3 in HLA-A2 CAR-Tregs improved Treg stability under inflammatory conditions without compromising suppressive function (251). Foxp3 is not the only possible target, as JNK1-deficient Tregs had improved survival as well as increased Lag-3 expression and IL-10 production compared to wildtype Tregs in a murine islet allograft model (252). A more complete understanding of the mechanisms of Treg function and stability has the potential to reveal genetic targets to improve Treg stability, survival, and long-term function.

An additional concern in Treg therapy, specifically in the *ex vivo* expansion of Tregs, is that attaining sufficient numbers of usable donor-specific Tregs is difficult, and some patients may not be able to produce an infusible Treg product (232). For this reason, the feasibility of Treg adoptive transfer therapy may be reliant on the ability to use allogeneic “off the shelf” Tregs. The obvious drawback of this approach is that these Tregs may be rapidly eliminated by alloreactive T cells. The logical solution of eliminating MHC expression from these third-party Tregs has the caveat of rendering them vulnerable to deletion by host NK cells activated by missing-self MHC (253–255). Various strategies have been attempted to protect edited cells from NK-mediated deletion including overexpression of the “don't-eat-me signal” CD47 (256), retention of a single HLA-C allele (257), or expression of HLA-E (258). Further progress in this field is likely needed before allogeneic Tregs can be safely and effectively harnessed to promote transplant tolerance.

In previously sensitized hosts, donor-specific CAR Tregs lost their ability to prolong graft survival and protect against production of donor specific antibodies, potentially due to anti-HLA antibodies blocking HLA on the graft and thus preventing CAR-Treg binding and activation, or to the reduced sensitivity of memory T cells to Treg-mediated suppression (224). The lack of efficacy in sensitized recipients is a particularly important consideration for clinical efficacy of CAR-Tregs since sensitization, due to pregnancy prior to transplantation, to transfusions, heterologous immunity, or prior transplantation, impacts a substantial proportion of transplant candidates. Over 10% of candidates on the U.S. waiting list have previously received transplants and up to 30% of candidates on the kidney transplant waiting list are considered highly sensitized (259, 260).

Another limitation is that Tregs may not induce true donor-specific tolerance, but merely prolongation of graft survival. One study of murine skin allograft recipients, in which endogenous Tregs were expanded *in vivo* using IL-2/anti-IL-2 complexes, found that although grafts were accepted long term, the induction of donor-specific tolerance did not occur, as 2nd donor-matched grafts were rejected (261). The absence of donor-specific tolerance implies reduced quality or quantity of redundant mechanisms maintaining acceptance of the first graft, such that transplant recipients may have to remain on permanent immunosuppressive therapies with possible consequent side effects and reduced quality of life. Thus, novel therapies may need to combine approaches to both augment the function of donor-specific Tregs and also tolerize conventional naïve and memory donor-reactive T and B cells to achieve universal and durable donor-specific transplantation tolerance.

In summary, some key aspects about Tregs in transplantation have emerged. First, is the ratio of Tregs:Tconvs in an allograft. This is associated with better graft outcome, relies on the expansion, recruitment, and induction of Tregs (85–89) and may be facilitated by DCs (93, 94, 99–101). Indeed, Foxp3 regulates both CCR4 and CCL3 to modulate Treg recruitment to the allograft and Treg proximity to Tconvs (41, 90, 96) and CCR4-mediated recruitment to the graft is driven by CCL22 produced by DCs and macrophages (90, 96). Second, is the phenotype of Tregs present in the graft as it is critical for Treg suppression of graft-infiltrating Tconvs. Increased expression of the inhibitory receptor PD-1 and tolerogenic cytokines TGFβ and IL-10 have been observed in Tregs in tolerance (85, 88, 92, 95). CTLA-4 expression on Tregs is important for both stripping CD80/CD86 from APCs via trogocytosis (43–46) and for triggering IDO expression by DCs which promotes Treg expansion and inhibits Tconv survival (47, 51). The role of Tregs as an IL-2 sink may be a key factor for Tconv suppression in allograft tolerance and down-regulation of Satb1 in Tconvs observed during tolerance leads to lower CD25 expression by Tconvs and better IL-2 competition by Tregs (189). Third, the therapeutic potential of Treg transfer is evident but the optimal way to harness the product to improve transplant outcomes is not yet clear. Donor-specific Tregs have greater efficacy in promoting graft survival, but polyclonal Tregs are easier to expand *ex vivo* (206–210, 213, 214). Furthermore, the use of both Tregs with direct and indirect allospecificity, expanded with F1 APCs, is more effective at promoting both acute and chronic graft survival than the use of Tregs with only direct allospecificity (207, 208, 221, 222). The use of CAR-Tregs allows their targeting to the graft and CAR-Tregs targeting HLA-A2 have been developed and tested in mouse models, and are currently in clinical trials (225–228, 240, 241). The efficacy of Treg transfer as a therapeutic is dependent on long-term stability, which may be improved by culture with rapamycin and vitamin C (230), on efficacy of suppression of memory alloreactive T cells, and on still reaching their antigenic target in sensitized hosts. Finally, as discussed above, many currently used immunosuppression therapies, including belatacept, basiliximab, and calcineurin inhibitors, deplete, destabilize, or otherwise inhibit Tregs. Interestingly, rapamycin seems much more permissive of Treg function and survival than other clinically used immunosuppressants. Thus, the impact of immunosuppressants on Tregs (endogenous or transferred) needs to be considered post-transplantation to allow Tregs the best opportunity to promote graft survival and function.
